# P02-022 - Atypical cryopirin associated periodic syndrome

**DOI:** 10.1186/1546-0096-11-S1-A129

**Published:** 2013-11-08

**Authors:** S Bujan Rivas, JI Arostegui Gorospe, A Sellas Fernandez, CP Simeon Aznar, J Aguilar Company, J Ordi Ros, M Vilardell Tarres

**Affiliations:** 1 Internal Medicine, Hospital Vall Hebron, Barcelona, Spain; 2 Immunology, Hospital Clinic I Provincial, Barcelona, Spain; 3 Rheumatology, Hospital Vall Hebron, Barcelona, Spain

## Introduction

Cryopyrin-associated periodic syndromes (CAPS) are dominantly inherited autoinflammatory diseases (AD) caused by NLRP3 mutations. They include different phenotypes (FCAS, Muckle-Wells syndrome, and CINCA/NOMID) with different severity, usually as childhood onset fever and urticarial-like rash. In the last years, the clinical picture of CAPS is growing with other manifestations than fever, urticaria, or sensorineural deafness.

## Objectives

To communicate the clinical picture, genetic mutation, therapeutic approach and follow-up of five patients from three generations of the same pedigree with late symptoms onset (at fourth decade of life) diagnosed from the study of urinary bladder AA-amyloidosis of the oldest member.

## Methods

Review of clinical file of the 5 involved patients from diagnosis until January 2013. Data studied included: onset and diagnosis age, past medical history, clinical, laboratory, pathologic and genetic data, treatment onset, clinical and laboratory evolution and adverse effects related to therapy.

## Results

71 yo. male was referred to Internal Medicine Service of Vall Hebron Hospital in June 2010 due to nephrotic syndrome and haemorraghic cystitis. A urinary bladder and rectal biopsies showed Congo-red deposits stained with antiamyloid A antibody. Patient’s medical history revealed self-limited episodic palindromic monoarthritis, and sensorineural hypoacusia since mid-thirties without recurrent fever or skin rash. ESR, CRP and serum A-amyloid protein (SAA) were elevated. Genetic study for AD identified a p.Ala-439-Thr NLRP3 mutation. Four family members, -3 adults (2 ♂/1♀) and 1 child (12 yo ♀)-, shared this mutation. All 3 adults presented adult-onset sensorineural deafness and SAA elevation; the two males also refered recurrent pericarditis and monoarthritis from mid-twenties on. Off-label treatment with rIL-1RA anakinra for symptomatic patients was considered a cost-effective option to canakinumab, the approved drug for CAPS. Early clinical remission, normalization of SAA, ESR and CRP and deafness stability was achieved. Index case 24-h proteinuria 20 months after anakinra was <500 mg. Anakinra adverse effects were minor local reaction at injection site (all patients) and transient alopecia in 1 adult female patient. After 24 months of anakinra, patients are asymptomatic. The child also remains asymptomatic since diagnosis.

## Conclusion

CAPS spectrum is still in evolution. These patients' phenotype was remarkable by late disease onset, reactive AA amyloidosis as haemorraghic cystitis and recurrent palindromic arthritis and pericarditis without urticarial-like skin rash or fever. Otherwise, anakinra proved to be an effective treatment in this CAPS serie. CAPS must be kept in mind for familiar sensorineural loss with AA amyloidosis and/or relapsing pericarditis or arthritis, even in adults.

## Disclosure of interest

None declared.

